# Clinical characteristics of peripherally inserted central catheter-related complications in cancer patients undergoing chemotherapy: a prospective and observational study

**DOI:** 10.1186/s12885-023-11413-0

**Published:** 2023-09-22

**Authors:** Ruixia Liu, Huiqiong Xu, Lihui Pu, Xiaofeng Xie, Hongxiu Chen, Zhoupeng Wu, Huirong Chen, Xiaoxia Zhang

**Affiliations:** 1 Department of Nursing, West China Hospital, Sichuan University / West China School of Nursing, Sichuan University, No.37 Guo Xue Street, PO Box 610041, Chengdu, Sichuan Province P.R. China; 2 Division of Abdominal Multimodality Treatment, Cancer Center, West China Hospital, Sichuan University / West China School of Nursing, Sichuan University, No.37 Guo Xue Street, PO Box 610041, Chengdu, Sichuan Province P.R. China; 3 Menzies Health Institute & School of Nursing and Midwifery, Griffith University, Brisbane Queensland, Australia; 4 Griffith University, Nathan Campus, Brisbane Queensland, PO Box 4111 Australia; 5 Innovation Center of Nursing Research, Nursing Key Laboratory of Sichuan Province, West China Hospital / West China School of Nursing, Sichuan University, No.37 Guo Xue Street, PO Box 610041, Chengdu, Sichuan Province P.R. China; 6 Division of Head & Neck Tumor Multimodality Treatment, Cancer Center, West China Hospital, Sichuan University, No.37 Guo Xue Street, PO Box 610041, Chengdu, Sichuan Province P.R. China; 7 Department of Vascular Surgery, West China Hospital, Sichuan University, No.37 Guo Xue Street, PO Box 610041, Chengdu, Sichuan Province P.R. China; 8 Innovation Center of Nursing Research, Nursing Key Laboratory of Sichuan Province, West China Hospital, No.37 Guo Xue Street, PO Box 610041, West, Chengdu, Sichuan Province P.R. China

**Keywords:** Cancer patients, Chemotherapy, Peripherally inserted central catheter, Catheter-related complications

## Abstract

**Purpose:**

The incidence of peripherally inserted central catheter (PICC)-related complications is higher in cancer patients than in noncancer patients. However, the pattern of specific complication occurrence over time remains unclear. The purpose of this study was to investigate the clinical characteristics of PICC-related complications in cancer patients undergoing chemotherapy.

**Methods:**

This prospective, observational study was conducted at a university-affiliated hospital in Western China. Cancer patients undergoing PICC insertion for anticancer treatment were recruited and followed up until the first week after catheter removal. Any complications, including occurrence time and outcomes, were recorded. The trajectory of specific PICC-related complications over time were identify based on the Kaplan‒Meier curve analysis.

**Results:**

Of the 233 patients analyzed, nearly half (n = 112/233, 48.1%) developed 150 PICC-related complication events. The most common were symptomatic catheter-related thrombosis (CRT) (n = 37/233, 15.9%), medical adhesive-related skin injury (MARSI) (n = 27/233, 11.6%), and catheter dislodgement (n = 17/233, 7.3%), accounting for 54.0% (n = 81/150, 54.0%) of total complications events. According to Kaplan‒Meier curve analysis, symptomatic CRT, pain, phlebitis, and insertion site bleeding were classified as the “early onset” group mainly occurring within the first month post-insertion. Catheter fracture and catheter-related bloodstream infection were classified as the “late onset” group occurring after the second month post-insertion. MARSI, catheter dislodgement, occlusion, and insertion site infection were classified as the “persistent onset” group persistently occurring during the whole catheter-dwelling period. Among the 112 patients with PICC-related complications, 50 (44.6%) patients had their catheters removed due to complications, and 62 (55.4%) patients successfully retained their catheters until treatment completion through conventional interventions. The major reasons for unplanned catheter removal were catheter dislodgement (n = 12/233, 5.2%), symptomatic CRT (n = 10/233, 4.3%), and MARSI (n = 7/233, 3.0%), accounting for 58.0% (n = 29/50, 58.0%) of the total unplanned catheter removal cases. Catheter dwelling times between patients with complications under successful interventions (130.5 ± 32.1 days) and patients with no complications (138.2 ± 46.4 days) were not significantly different (t = 1.306, *p* = 0.194; log-rank test = 2.610, *p* = 0.106).

**Conclusions:**

PICC-related complications were pretty common in cancer patients undergoing chemotherapy. The time distribution of PICC-related complications varied, and medical staff should develop time-specific protocols for prevention. Because more than half of the patients with PICC-related complications could be managed with conventional interventions, PICCs remain a priority for cancer patients undergoing short-term chemotherapy. The study was registered in 02/08/2019 at Chinese Clinical Trial Registry (registration number: ChiCTR1900024890).

**Supplementary Information:**

The online version contains supplementary material available at 10.1186/s12885-023-11413-0.

## Introduction

Peripherally inserted central catheters (PICCs) are used as first-line central venous catheters (CVCs) for the delivery of chemotherapy and supportive care in cancer patients under stable hemodynamic status [1, 2]. Compared to other types of CVCs, PICCs are inserted by nurse-led teams [1, 2] with short operating times [1] and low insertion costs [3, 4]. However, patients with PICCs can also suffer from a range of PICC-related complications leading to treatment interruption [5, 6], prolonged hospitalization [7], and increased costs [4, 8].

In the context of COVID-19, the incidence of PICC-related complications has increased [9, 10], but medical resources are more scarce [11, 12]. To make the best use of medical resources, medical staff need to assess the benefits and risks of PICCs before insertion. A comprehensive understanding of the clinical features of PICC-related complications is a prerequisite for medical staff to make the best decisions. However, in terms of onset features, most studies report the incidence rate of PICC-related complications at a specific time point, usually by the time of catheter removal [2, 13] or complication onset [14–16]. Studies have shown that the onset time of PICC-related complications varies [16–18]. However, the pattern of specific complication occurrence over time should be further explored [19]. Moreover, infusion therapy guidelines recommend various measures to manage PICC-related complications; however, because of the hierarchy of the recommended evidence [20, 21], the effectiveness of these measures remains to be further confirmed in the real clinical settings in cancer patients undergoing chemotherapy.

Therefore, we designed this time-continuous prospective study to outline the clinical characteristics of PICC-related complications in cancer patients undergoing chemotherapy, including the occurrence time and lasting span of PICC-related complications, as well as their outcomes.

## Methods

### Design and setting

This was a prospective, observational study with the primary purpose of investigating the pattern of occurrence and outcome of PICC-related complications. This study was conducted at the West China Hospital of Sichuan University, a 4500-bed university-affiliated hospital in western China.

### Ethical considerations

This study was registered in Chinese Clinical Trial Registry (registration number: ChiCTR1900024890). The study was approved by the Institutional Review Board of West China Hospital of Sichuan University [Approval number: 2019 (56)]. All patients provided written informed consent for research purposes.

### Participants

Cancer patients admitted to the cancer center who required at least one course of chemotherapy and scheduled for PICC insertion were included. The inclusion criteria were as follows: (1) age range of 18–80 years; (2) undergoing insertion of PICC for the first time; (3) expected survival time ≥ 6 months; and (4) conscious and able to communicate orally or in writing. The exclusion criteria were as follows: (1) other types of CVCs, e.g., dialysis catheters and totally implantable venous access ports (PORTs); and (2) catheter tips outside of the distal superior vena cava or cava atrium junction. The elimination criteria were as follows: (1) patient requests to remove the catheter, in the absence of any complication and (2) loss to follow-up.

### Research procedure

#### Patient approach

In our cancer center, patients scheduled for chemotherapy are routinely inserted with PICCs unless the patient is contraindicated for PICC insertion. Each patient was approached for the study when first admitted to the cancer center. After admission, general information about the study was given to patients by the nurse in charge. If the patient wanted to participate in the study, the patient would contact the nurse in charge of enrollment and would be given detailed information about the study in person. Subsequently, written informed consent was provided by the patients.

#### PICC insertion and maintenance

The type of catheter used in this study was a 4 F- or 5 F-Bard Power PICC (Bard Access Systems, Salt Lake City, UT, USA). PICCs were inserted into the upper arm vein by ultrasound using the modified Seldinger technique. After insertion, the catheter tip was verified using posteroanterior chest X-ray radiography. If the catheter tip was located in the distal superior vena cava, this was considered acceptable; otherwise, catheter tip adjustments were performed under aseptic conditions. Catheter maintenance was strictly performed following a nursing protocol, which was developed based on two infusion therapy guidelines (Infusion Therapy Standards of Practice 2016 version and Nursing Practice of Intravenous Therapy: Guideline and Implementation) [20, 21]. The key elements of catheter maintenance, including dressing change, changing the needleless connector, catheter flush and lock as well as upper extremity exercises, are shown in **supplementary Table **S1.

#### Definition and management of PICC-related complications

The diagnostic criteria and management measures of PICC-related complications were principally followed by the infusion therapy guidelines mentioned previously [20, 21]. The definition of PICC-related complications was presented in **supplementary Table **S2.

#### Data collection and follow-up

Demographic and clinical information on the patients were collected before insertion. After catheter insertion, regardless of whether patients participated in the study, they were routinely given a self-designed PICC maintenance manual by the nurse, the main content of which included catheter insertion, maintenance, and complication management. The catheter status was recorded in this manual by the nurse each time maintenance was performed. Catheter insertion-related information was completed by the nurses responsible for PICC insertion. Inpatient and outpatient care of the catheters was completed by inpatient and outpatient nurses, respectively. The endpoint of the follow-up was the first week after the catheter was removed, at when the patient would receive a follow-up interview by telephone.

### Statistical analysis

SPSS 26.0 software (IBM Corp., Armonk, NY, USA) was used to analyze the data. Descriptive statistics, such as frequencies and proportions, were used to describe the study sample. Kaplan‒Meier curves were used to estimate the cumulative incidence of PICC-related complications and unplanned catheter removal. The trajectory of specific PICC-related complications over time were identify based on the Kaplan‒Meier curve analysis. Catheter survival time was censored on the day during which a PICC was removed due to the completion of therapy or a complication. Independent sample t-test was used to compare the difference in catheter dwelling time between patients with complications under successful interventions and patients with no complications.

## Results

### Study procedures

**Fig. 1:**
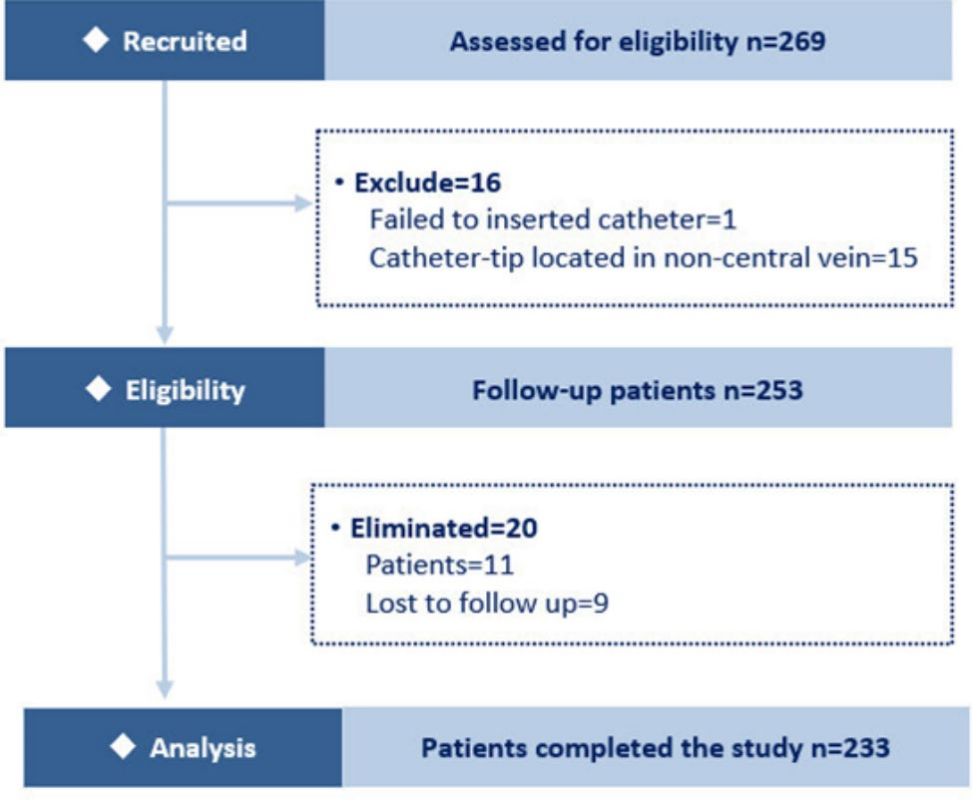
Study flow diagram

This study was conducted from May 2019 to July 2020. Of the 269 patients recruited, 36 were excluded or eliminated, and 233 patients with complete data were included in the final analysis (Fig. 1). The total cumulative duration of follow-up was 27,705 days, and the mean time was 118.9 days (range 3–302 days).

### Patient characteristics

Over half of the patients were male (n = 139, 59.7%), and the majority of the catheter types were 4 F (n = 153, 65.7%), principally inserted into the right upper arm (n = 147, 63.1%) and basilic veins (n = 203, 87.1%). The majority of patients (n = 198, 85.0%) received chemotherapy, while handful of patients (n = 35, 15.0%) received combined chemotherapy and immunotherapy. The more detail information involved patients’ characteristics as shown in Table 1.

**Table 1:** Patient characteristics (n = 233)

Characteristics	n (%)
Age (year, mean ± standard deviation)	52.5 ± 10.7
Sex	
Male	139 (59.7)
Female	94 (40.3)
Diagnosis	
Gastrointestinal cancer	66 (28.3)
Lung cancer	57 (24.5)
Head and neck Cancer	40 (17.2)
Breast cancer	27 (11.6)
Lymphoma	23 (9.8)
Other	20 (8.6)
Cancer stage	
I/II	46 (19.7)
III	103 (44.2)
IV	84 (36.1)
Barthel index (mean ± SD)	80.0 ± 10.0
Body mass index (mean ± SD)	23.2 ± 2.4
Treatment regime	
Chemotherapy	198 (85.0)
Chemotherapy and immunotherapy	35 (15.0)
Insertion vein	
Basilic vein	203 (87.1)
Other	30 (12.9)
Insertion arm	
Left	86 (36.9)
Right	147 (63.1)
PICC gauge	
4 F	153 (65.7)
5 F	80 (34.3)
Complication rate	
Symptomatic CRT	37 (15.9)
MARSI	27 (11.6)
Catheter dislodgment	17 (7.3)
Occlusion	16 (6.9)
Pain	13 (5.6)
Insertion site infection	12 (5.2)
Phlebitis	11 (4.7)
Catheter fracture	9 (3.9)
Insertion site bleeding	6 (2.6)
CLABSI	2 (0.9)

CRT: catheter-related thrombosis; MARSI: medical adhesive-related skin injury; CLABSI: catheter-related bloodstream infection

### Clinical features of PICC-related complications

#### PICC-related complications calculated by patient case

**Fig. 2:**
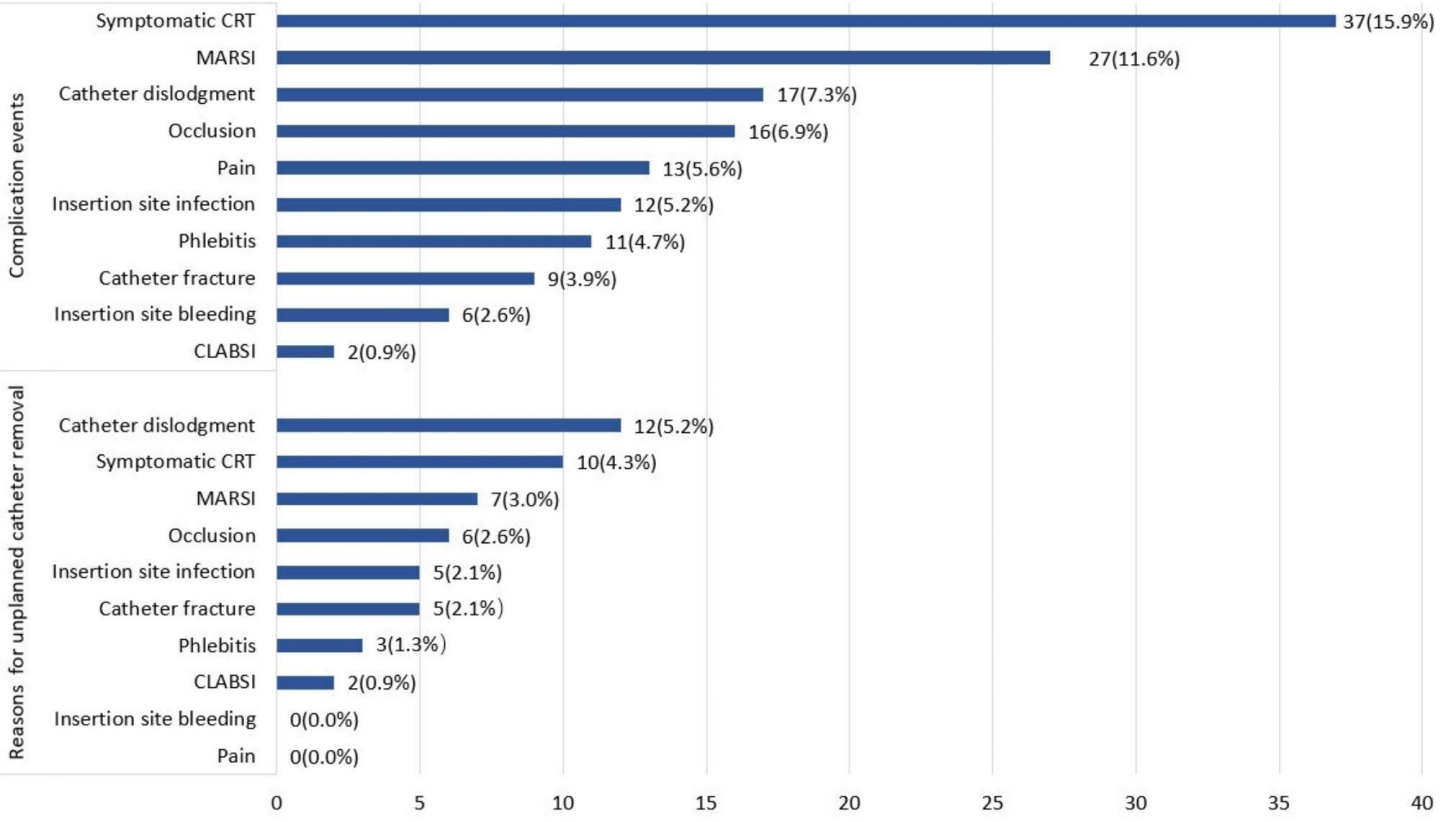
The frequency of complication events and reasons for unplanned catheter removal. CRT: catheter-related thrombosis; MARSI: medical adhesive-related skin injury; CLABSI: catheter-related bloodstream infection

Of the 233 patients analyzed, 112 (48.1%) developed 150 PICC-related complication events. Seventy-five patients presented with 1 complication, 30 presented with 2 complications, and 5 presented with 3 complications. The major complications were symptomatic catheter-related thrombosis (CRT) (n = 37, 15.9%), medical adhesive-related skin injury (MARSI) (n = 27, 11.6%), and catheter dislodgement (n = 17, 7.3%), accounting for 54.0% (n = 81/150) of the total complication events (Fig. 2).

#### PICC outcomes calculated by patient case

Of the 112 patients with PICC-related complications, 50 patients (44.6%) had their catheters removed due to complications; 62 patients (54.4%) successfully retained their catheters until the completion of anticancer treatment via conventional interventions. The most common reasons for unplanned catheter removal were catheter dislodgement (n = 12, 5.2%), symptomatic CRT (n = 10, 4.3%), and MARSI (n = 7, 3.0%), accounting for 58.0% (n = 29/50) of the total cases of unplanned catheter removal (Fig. 2). Catheter dwelling time between patients with complications under successful interventions (130.5 ± 32.1 days) and patients with no complications (138.2 ± 46.4 days) was not significantly different (t = 1.306, *p* = 0.194; log-rank test = 2.610, *p* = 0.106), as shown in Fig. 3a and b.

**Fig. 3a:**
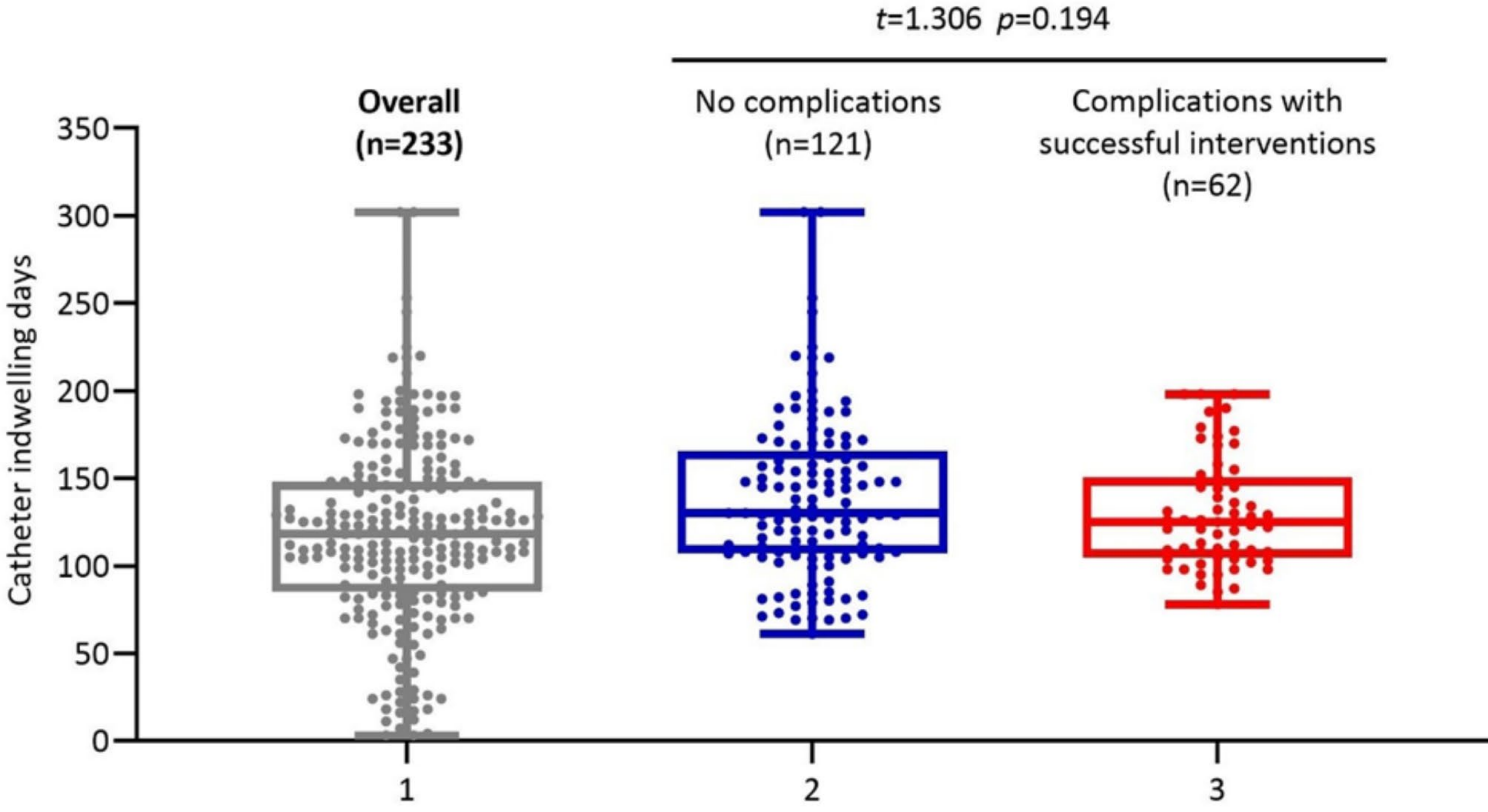
Comparison of catheter dwelling time

**Fig. 3b:**
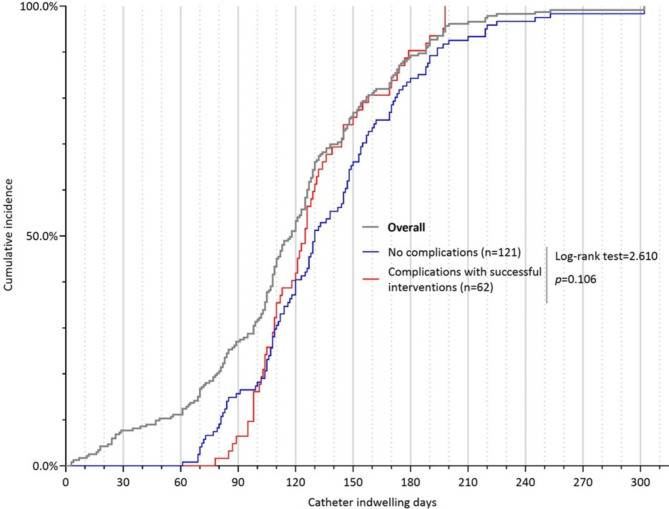
Kaplan‒Meier curve of catheter indwelling time

#### Time distribution of PICC-related complications

According to Kaplan‒Meier curve analysis, symptomatic CRT (n = 37, 15.9%), pain (n = 13, 5.6%), phlebitis (n = 11, 4.7%), and insertion site bleeding (n = 6, 2.6%) were classified as the “early onset” group mainly occurring within the first month post-insertion. Catheter fracture (n = 9, 3.9%) and catheter-related bloodstream infection (CLABSI) (n = 2, 0.9%) were classified as the “late onset” group occurring after the second month post-insertion. MARSI (n = 27, 11.6%), catheter dislodgement (n = 17, 7.3%), occlusion (n = 16, 6.9%), and insertion site infection (n = 12, 5.2%) were classified as the “persistent onset” group persistently occurring during the whole catheter-dwelling period. The cumulative incidence of complications is detailed in Fig. 4a and b.

**Fig. 4a:**
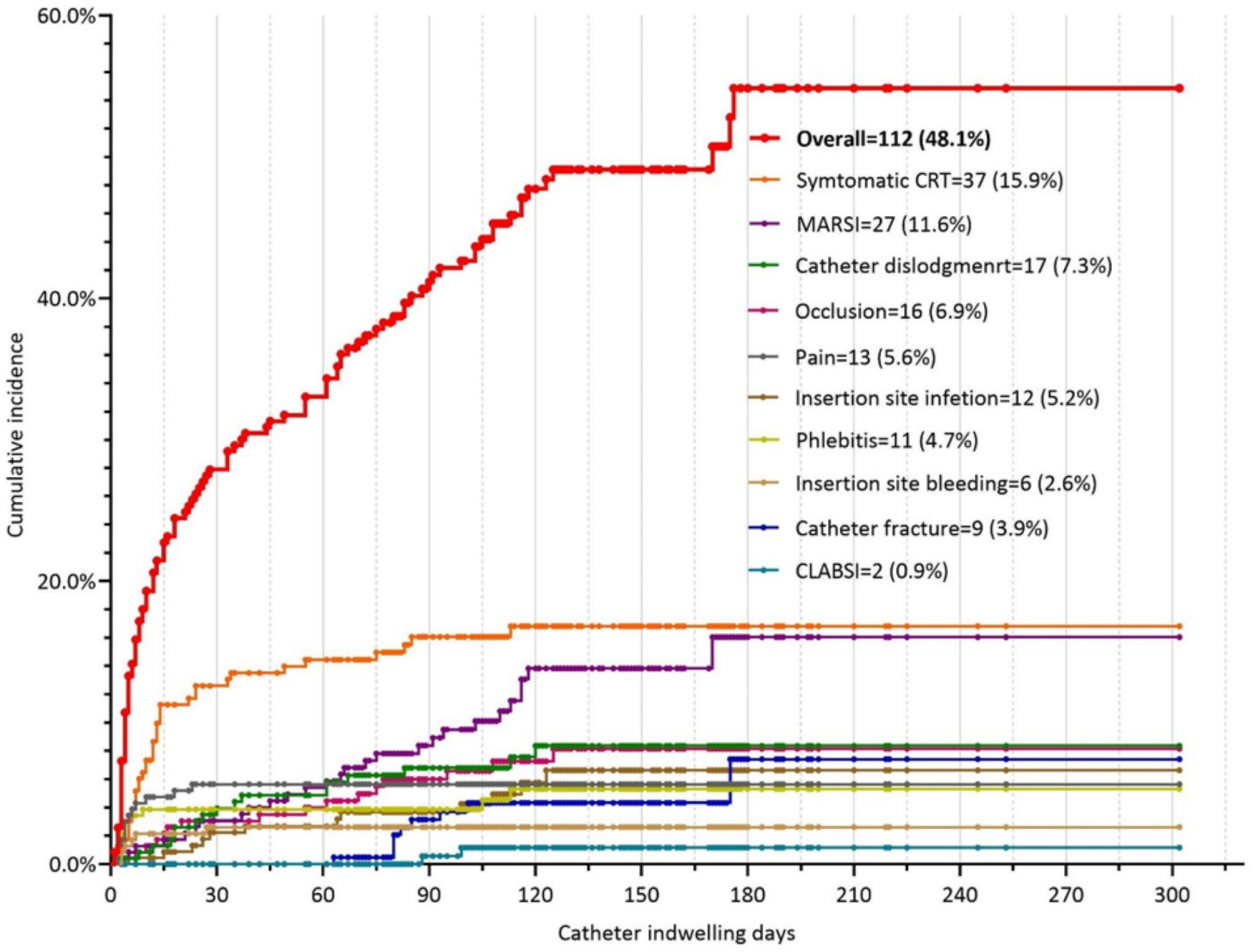
The cumulative incidence of PICC-related complications. CRT: catheter-related thrombosis; MARSI: medical adhesive-related skin injury; CLABSI: catheter-related bloodstream infection. Each point represents a censored patient

**Fig. 4b:**
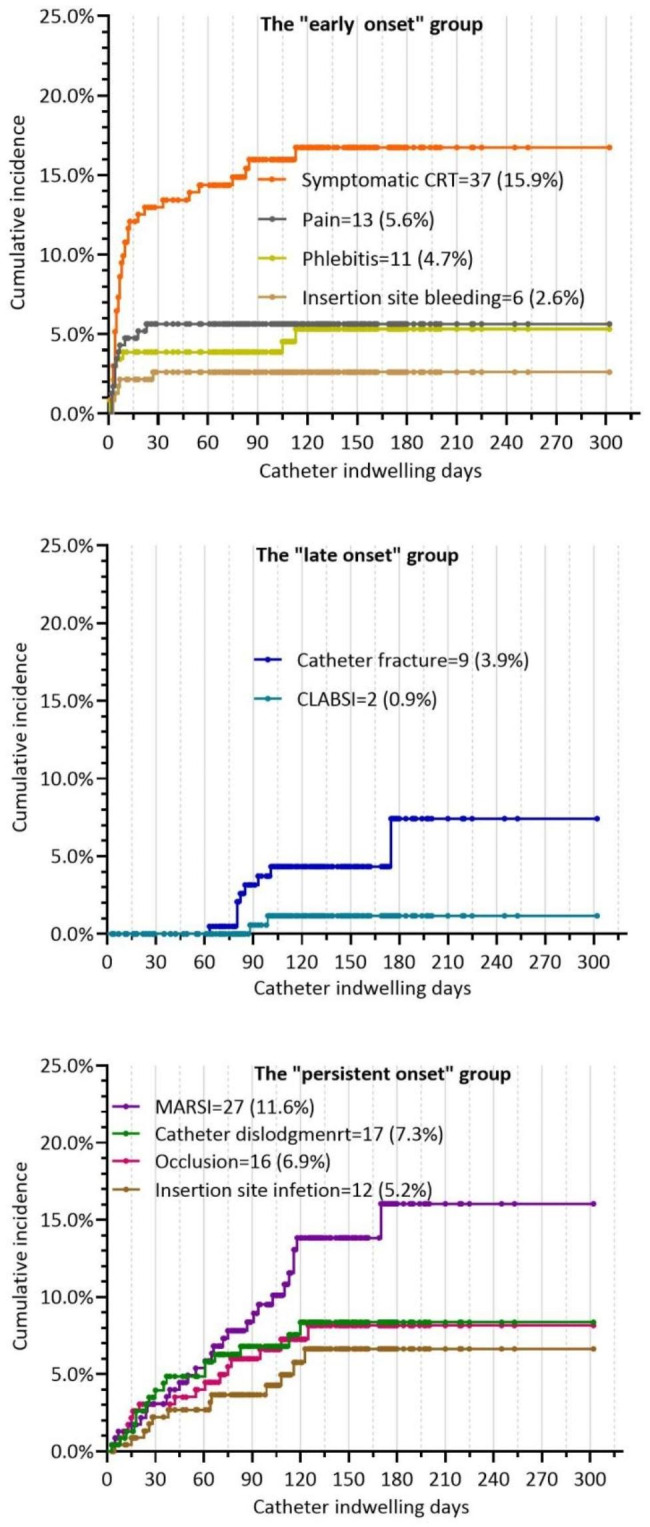
Three groups of time distribution of PICC-related complications. CRT: catheter-related thrombosis; MARSI: medical adhesive-related skin injury; CLABSI: catheter-related bloodstream infection. Each point represents a censored patient

#### Time distribution of unplanned catheter removal

According to Kaplan‒Meier curve analysis, unplanned catheter removal because of catheter fracture (n = 5, 2.1%) and CLABSI (n = 2, 0.9%) were classified as the “late removal” group occurring after the second month post-insertion. All other complications caused by unplanned catheter removal were classified as the “persistent removal” group persistently occurring during the whole catheter-dwelling period, including catheter dislodgement (n = 12, 5.2%), symptomatic CRT (n = 10, 4.3%), MARSI (n = 7, 3.0%), and catheter occlusion (n = 6, 2.6%). The cumulative incidence of unplanned catheter removal is shown in Fig. 5a and b.

**Fig. 5a:**
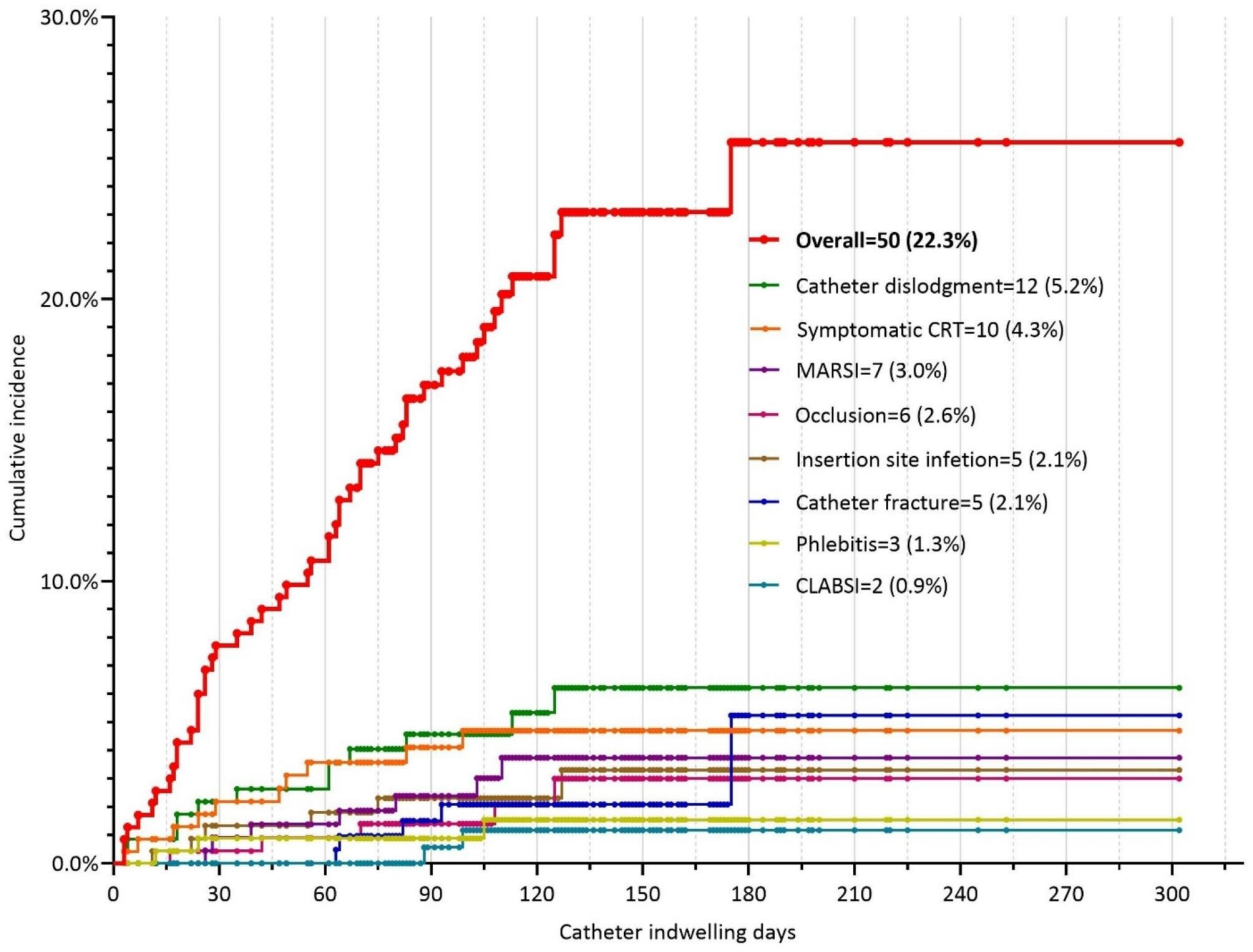
The cumulative incidence of unplanned catheter removal. CRT: catheter-related thrombosis; MARSI: medical adhesive-related skin injury; CLABSI: catheter-related bloodstream infection. Each point represents a censored patient

**Fig. 5b:**
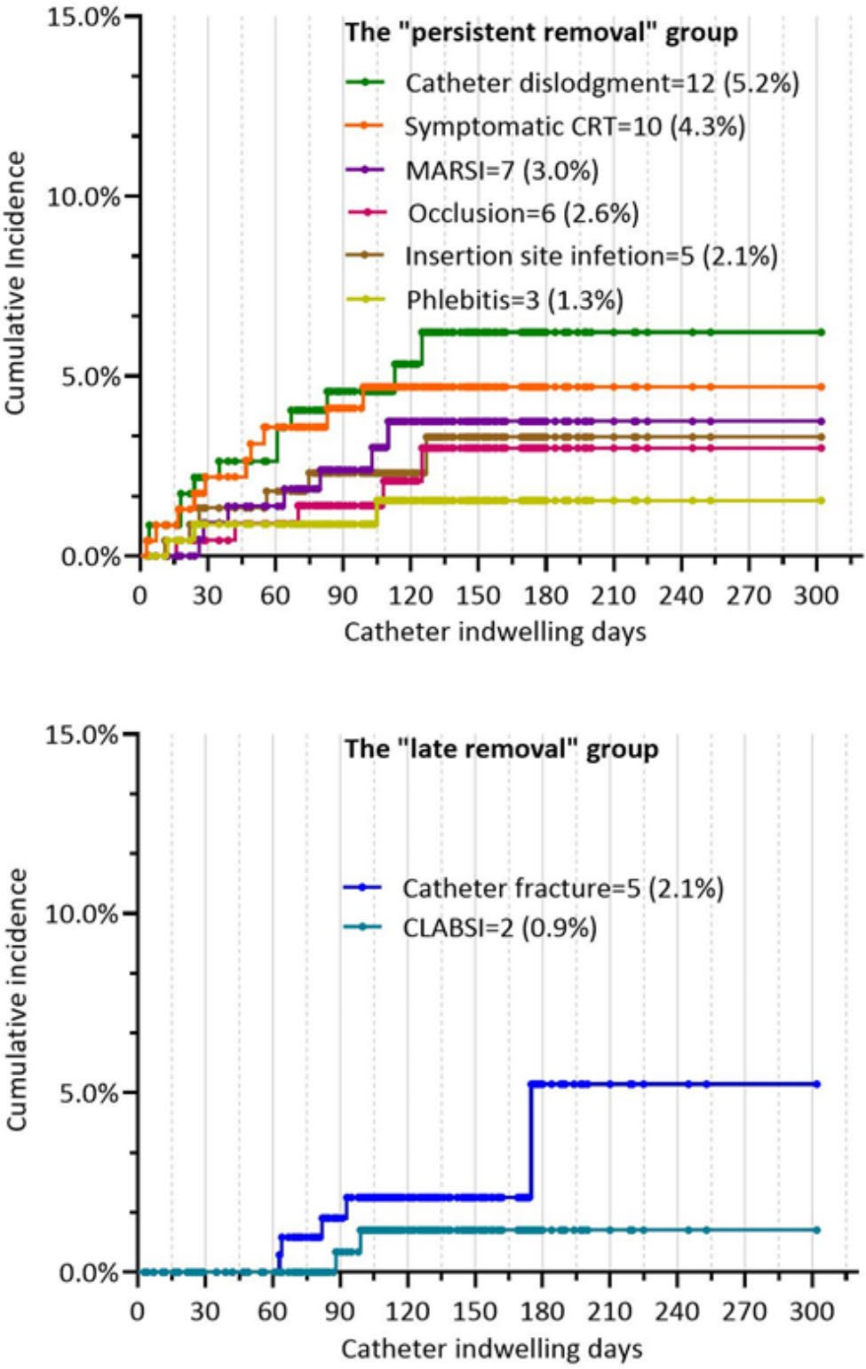
Two groups of time distribution of unplanned catheter removal. CRT: catheter-related thrombosis; MARSI: medical adhesive-related skin injury; CLABSI: catheter-related bloodstream infection. Each point represents a censored patient

## Discussion

Our findings indicate that PICC-related complications involved approximately half of cancer patients undergoing chemotherapy. In terms of onset features, PICC-related complications show three groups of time distributions (early, late, and persistent onset). Additionally, more than half of the patients with complications successfully retained their catheters until completion of anticancer treatment via conventional interventions.

### High rate of PICC-related complications

PICCs play a vital role in anticancer treatment for cancer patients. However, the incidence of PICC-related complications was higher in cancer patients than in noncancer patients [22]. Previous studies reported that the incidence of PICC-related complications in cancer patients range from 14.4 to 67.3% [1, 2, 7, 23–25], whereas, in our study, nearly half of patients (n = 112, 48.1%) experienced PICC-related complications, and is higher than most of the published series to date. We suggest that multiple reasons mainly account for this finding. Unlike similar studies, our study used an open observational approach and collected all complications between catheter insertion and the first week after catheter removal, which included some complications excluded from similar studies, such as pain and MARSI. The unique method of observation may have contributed to the higher rate of PICC-related complications in our study. Studies report that BMI > 25 and advanced cancer are risk factor for PICC-related complications [13, 26]. In our study, almost half of the patients with BMI > 25 and two-thirds diagnosed with advanced stage cancer, which also substantially increased the risk of PICC-related complications.

Of all the complication types that were monitored, symptomatic CRT was the most frequent (n = 37, 15.9%). This is related to the characteristics of the PICC device, including the smaller diameter of the inserted vein [2], longer length of the catheter [27], frequent movement of the elbow and the upper arm [28], etc. MARSI, another complication that was observed with a high incidence in our study (n = 27, 11.6%), has attracted limited attention compared to symptomatic CRT. MARSI is worthy of attention, as studies have reported that this complication occurs in up to 29.8% of cancer patients with PICCs [29–31]. Additionally, MARSI adds approximately £1.10 to £7.90 per patient and requires 1–8 weeks to treat [32]. Our results showed that the incidence of MARSI was 11.6% (n = 27), of which 7 (3.0%) patients had to have their catheters removed. Therefore, we suggest that more attention be given to preventing MARSI.

### Time distributions of PICC-related complications

There is a lack of standardized definitions for early and late complications, and there is very limited relevant literature available. In Corti et al., complications that occurred ⩽60 days from catheter insertion were considered early complications; adverse events registered after 60 days from positioning of the device were regarded as late complications [33]. We refer to the definitional approach of Corti et al., in classifying complications, but the more important basis is the time distribution of PICC-related complications in Kaplan‒Meier curve analysis. This study shows that there are three groups of time distributions for PICC-related complications, i.e., the “early onset” group (within one month), the “late onset” group (after two months), and the “persistent onset” group (through the whole catheter dwelling period).

From a pathogenesis perspective, the four early-onset complications, i.e., symptomatic CRT, phlebitis, pain, and insertion site bleeding, are associated with the loss of vessel integrity and mechanical damage to vessel caused by PICC insertion. Phlebitis and symptomatic CRT can be considered different phases of the same pathological change [34], and are both accompanied by local pain and insertion site bleeding. The alignment with histopathology accounts for the concentrated occurrence of the above-mentioned complications.

Many studies have demonstrated that the dwelling time of catheters remains an important risk factor for CLABSIs, but the threshold time depends on the patient’s specific systemic status. For example, He et al., reported that bone tumor patients with PICCs for more than 30 days had 4.2 times more CLABSIs than bone tumor patients with PICCs inserted for less than 30 days [35]. Pitiriga et al., reported that the incidence of CLABSI was 12.0 times higher in hospitalized patients with CVCs inserted for more than 20 days than in those patients with CVCs inserted for less than 10 days [36]. Apart from catheter indwelling time, studies have reported that patients undergoing chemotherapy experience 4.5–11.4 times more CLABSIs than patients undergoing total parenteral nutrition or intravenous infusion [37, 38]. Although CLABSI occurred in our study after the second month post-insertion, only two patients (0.90%) developed a CLABSI. Therefore, our study did not achieve a confidence estimate for CLABSI the threshold time.

### Catheters with complications under successful interventions with no impact on catheter indwelling time

Of the 112 patients with PICC-related complications, 50 (44.6%) had their catheters removed due to complications, and 62 (54.4%) successfully retained their catheters until completion of anticancer treatment via conventional interventions. The major reasons for unplanned catheter removal were catheter dislodgement (n = 12, 5.2%), symptomatic CRT (n = 10, 4.3%) and MARSI (n = 7, 3%), accounting for 58.0% (n = 29/50, 58.0%) of the total cases of unplanned catheter removal. Studies have also reported catheter dislodgement, symptomatic CRT, and MARSI as the major causes of unplanned catheter removal [13]. For patients with complications under successful interventions, their catheter indwelling time (130.5 ± 32.1 days) was similar to that of patients with no complications (138.2 ± 46.4 days) (t = 1.306, *p* = 0.194; log-rank test = 2.610, p = 0.106). Regarding the interventions used to address complications, we did not adopt a complex approach, and nearly half of patients (n = 46/112, 46.4%) with complications successfully retained their catheters by local and non-invasive managements. Four randomized controlled trials and three meta-analyses and systematic reviews compared PORTs with PICCs. The findings of these studies suggest that PORTs might be superior in terms of complication rates (OR = 0.50–0.64) [1, 2, 5, 6, 39] and unplanned catheter removal rates (OR = 0.12–0.49) [2, 5, 6, 28, 29, 39]. However, once complications require the removal of the catheter, PICCs are more convenient to intervene than PORTs. Additionally, Shao et al., and Wang et al., compared the average total cost between PORTs and PICCs and found a much lower average total cost for patients with PICCs within 6 months (PICC vs. PORTs=¥ 4091.7 vs. ¥ 4566.8) [8] and within 9 months (PICCs vs. PORTs=$731.4 vs. $1414.48) [4]. Therefore, PICCs remain advantageous for cancer patients scheduled to undergo short-term chemotherapy in terms of ease of insertion and removal as well as average total cost.

### Limitations

Our study also has limitations. First, we may overestimate the incidence of PICC-related complications. In our study, the incidence of PICC-related complications was higher than most of the published literature to date, except for the study by Simonetti et al., which showed that the incidence of PICC-related complications was 67.3% in neuro-oncological patients [23]. Studies have shown that patients with active cancer have a higher incidence of PICC-related complications, from 22.4 to 52.0% [1, 2, 6], while patients with non-active cancer have a comparatively lower incidence, from 13.0 to 18.5% [40–42]. In our study, all patients were receiving chemotherapy, two-thirds had advanced cancer, and their status was considered active cancer, which may account for the higher incidence of PICC-related complications than observed in most similar studies. Second, the small sample size of our study resulted in a low incidence of CLABSI (n = 2, 0.9%). Therefore, we were unable to confidently estimate the precise threshold time of catheter days to avoid CLABSI. A study conducted by Park et al., which included 1053 mixed patients, found that the threshold time for CLABSI was 25 days in patients with PICCs [17]. Because of the abundant cases in the Park et al., study, we suggest the threshold time for CLABSI in patients with PICCs, after the third week post-insertion, could be adopted as a reference. In addition, future studies with larger sample sizes are needed to determine the time distribution of CLABSI in cancer patients undergoing chemotherapy using PICCs.

Although our study has the above limitations, by using a prospective study design and multi-demonstration of PICC-related complications, we believe these limitations do not detract from the main purpose of investigating the clinical features of PICC-related complications.

## Conclusion

The incidence of PICC-related complications was quite high in cancer patients undergoing chemotherapy. The pattern of PICC-related complication occurrence over time varied, which suggests that medical staff develop time-specific protocols for prevention. Because more than half of patients with PICC-related complications could be managed under conventional interventions, PICCs remain a priority for cancer patients undergoing short-term chemotherapy.

### Electronic supplementary material

Below is the link to the electronic supplementary material.

**Table S1**. The key elements of catheter maintenance. **Table S2**. The definition of PICC-related complications.

## Data Availability

The data that support the findings of this study are available from the corresponding author upon reasonable request.
