# Supplementary material for: Clinical characteristics of peripherally inserted central catheter-related complications in cancer patients undergoing chemotherapy: a prospective and observational study
Source: BMC Cancer. 2023 Sep 22;23:894. doi: 10.1186/s12885-023-11413-0 (PMC10515037; doi:10.1186/s12885-023-11413-0)
Supplement: Supplementary file 1 — Table S1. The key elements of catheter maintenance. Table S2. The definition of PICC-related complications. [file 12885_2023_11413_MOESM1_ESM.docx]

**Table S1.** The key elements of catheter maintenance.

| Items | Elements |
| --- | --- |
| Dressing change | - the first dressing change was applied approximately 24 hours post-insertion, or immediately if the integrity of the dressing became compromised (e.g., visibly soiled, presence of moisture, drainage, or blood) or if compromised skin integrity was present under the dressing; - dressing changes were routinely conducted once a week. |
| Changing the needleless connector | - the needleless connector was changed at least once a week, immediately after blood or lipid infusion, etc. |
| Catheter flush and lock | - catheter flush and lock with prefilled 10 ml normal saline syringes was performed using the pulsatile method; - catheter flush and lock was performed at least once a week and immediately before and after every intravenous drug delivery. |
| Upper limb exercises | - exercises such as handgrip, wrist joint rotation, and elbow flexion/extension were performed at least 3–5 times/day in sets of 5–10 minutes. |
| Catheter maintenance address | - during the patient admission to cancer center for chemotherapy, the inpatient nurses were responsible for catheter maintenance; - during chemotherapy intervals, patients attended the PICC clinic in our hospital and the outpatient nurse were responsible for catheter maintenance. |

**Table S2.** The definition of PICC-related complications[1, 2].

| Complication | Definition |
| --- | --- |
| Symptomatic catheter-related thrombosis | Arm at the inserted side with oedema, swelling and pain and ultrasonic diagnosis indicates that there is thrombosis within relevant vein |
| Pain | Spontaneous mention by the patient of significant and persistent pain |
| Phlebitis | According to the revised grade standards of phlebitis made by American INS in 2016, clinical manifestations such as pain, erythema, oedema and palpable venous cord |
| Insertion site bleeding | The site of insertion still bleeding over 72 h after PICC insertion |
| Catheter fracture | Some liquids may leak from the catheter or the catheter fails to realize its function out of its completed broken situation |
| Catheter-related bloodstream infection | Isolation of the same micro-organism in peripheral blood and PICC  cultures |
| Medical adhesive-related skin injury | Redness, tears, or erosion of the skin, or development of vesicles or bulla in an area exposed to medical adhesive and lasting for 30 minutes or more following adhesive removal |
| Catheter dislodgement | Accidental removal or movement of the catheter |
| Occlusion | The inability to infuse medication or blood through the PICC in the absence of line malposition |
| Insertion site infection | The presence or purulent discharge with erythema and/or tenderness close to the catheter exit site. |

1. Gorski LA: **The 2016 Infusion Therapy Standards of Practice**. *Home healthcare now* 2017, **35**(1):10-18.

2. Wang JRC, M.,, Hu B: **Nursing practice of intravenous therapy: guideline and implementation. Military Science Publishing House**. BeijinMilitary Science Publishing House; 2010.
